# Cytochrome P450 Metabolism of Betel Quid-Derived Compounds: Implications for the Development of Prevention Strategies for Oral and Pharyngeal Cancers

**DOI:** 10.1155/2013/618032

**Published:** 2013-08-01

**Authors:** Che-Yi Lin, Tien-Szu Pan, Chun-Chan Ting, Shih-Shin Liang, Shu-Hung Huang, Hsiu-Yueh Liu, Edward Cheng-Chuan Ko, Chung-Wei Wu, Jen-Yang Tang, Ping-Ho Chen

**Affiliations:** ^1^Department of Oral and Maxillofacial Surgery, Chi Mei Hospital, Liouying 736, Taiwan; ^2^Department of Electronic Engineering, National Kaohsiung University of Applied Sciences, Kaohsiung 807, Taiwan; ^3^School of Dentistry, College of Dental Medicine, Kaohsiung Medical University, Kaohsiung 807, Taiwan; ^4^Department of Periodontology, School of Dentistry, Aichi Gakuin University, Nagoya 464-8651, Japan; ^5^Department of Biotechnology, Kaohsiung Medical University, Kaohsiung 807, Taiwan; ^6^Center for Resources, Research and Development, Kaohsiung Medical University, Kaohsiung 807, Taiwan; ^7^Graduate Institute of Medicine, College of Medicine, Kaohsiung Medical University, Kaohsiung 807, Taiwan; ^8^Division of Plastic Surgery, Department of Surgery, Kaohsiung Medical University Hospital, Kaohsiung Medical University, Kaohsiung 807, Taiwan; ^9^Department of Dental Technology, Shu Zen College of Medicine and Management, Kaohsiung 807, Taiwan; ^10^Division of Oral and Maxillofacial Surgery, Kaohsiung Medical University Hospital, Kaohsiung 807, Taiwan; ^11^Departments of Cartilage and Bone Regeneration (Fujisoft), Graduate School of Medicine, The University of Tokyo, Tokyo 113-8655, Japan; ^12^Department of Radiation Oncology, Faculty of Medicine, College of Medicine, Kaohsiung Medical University, Kaohsiung 807, Taiwan; ^13^Department of Radiation Oncology, Kaohsiung Medical University Hospital, Kaohsiung 807, Taiwan; ^14^Cancer Center, Kaohsiung Medical University Hospital, Kaohsiung Medical University, Kaohsiung 807, Taiwan

## Abstract

Betel quid (BQ) products, with or without tobacco, have been classified by the International Agency for Research on Cancer (IARC) as group I human carcinogens that are associated with an elevated risk of oral potentially malignant disorders (OPMDs) and cancers of the oral cavity and pharynx. There are estimated 600 million BQ users worldwide. In Taiwan alone there are 2 million habitual users (approximately 10% of the population). Oral and pharyngeal cancers result from interactions between genes and environmental factors (BQ exposure). Cytochrome p450 (*CYP*) families are implicated in the metabolic activation of BQ- and areca nut-specific nitrosamines. In this review, we summarize the current knowledge base regarding *CYP* genetic variants and related oral disorders. In clinical applications, we focus on cancers of the oral cavity and pharynx and OPMDs associated with *CYP* gene polymorphisms, including *CYP1A1*, *CYP2A6*, * CYP2E1*, and *CYP26B1*. Our discussion of *CYP* polymorphisms provides insight into the importance of screening tests in OPMDs patients for the prevention of oral and pharyngeal cancers. Future studies will establish a strong foundation for the development of chemoprevention strategies, polymorphism-based clinical diagnostic tools (e.g., specific single-nucleotide polymorphism (SNP) “barcodes”), and effective treatments for BQ-related oral disorders.

## 1. Introduction

Oral and pharyngeal cancers are some of the most common cancers worldwide [1]. Taiwan is a hyperendemic area for oral and pharyngeal cancers [2]. In 2010, the age-standardized incidence rate adjusted by 2000 years world population (ASRW) of oral and pharyngeal cancers was 40.56 per 100 000 Taiwanese males and was ranked the fourth most prevalent cancer in Taiwan [3]. The ASRW of oral and pharyngeal cancers among Taiwanese males was also ranked one of the highest worldwide [2].

Oral submucous fibrosis (OSF), leukoplakia, erythroplakia, and lichen planus are a group of oral potentially malignant disorders (OPMDs) thought to be linked to the development of oral and pharyngeal cancers [4, 5]. Cases of oral and pharyngeal cancers are concentrated in Southeast Asia, where betel quid (BQ) chewing is prevalent [2]. Habitual BQ chewing is associated with an increased risk of oral and pharyngeal cancers and OPMDs [5–9]. Ko et al. demonstrated a significant association between BQ chewing without tobacco and the incidence of oral cancer [6]. In addition, BQ chewing is a major risk factor for OPMDs such as oral leukoplakia and OSF, and cigarette smoking was shown to have a modifying effect on chewing, based on an additive-interaction model in oral leukoplakia patients [7].

The fourth most frequently consumed psychoactive substance worldwide after caffeine, nicotine, and alcohol is BQ, a masticatory mixture that combines the areca nut (AN), betel leaf, slaked lime, and various local flavorings [10]. Approximately 10% of the global population (approximately 600 million users) chew some form of BQ, primarily in the Indo-Pakistan subcontinent, South and Southeast Asia, and the South Pacific islands, and a large number of South Asian immigrants to the United Kingdom, Africa, Australia, and the United States are also BQ chewers [10–12]. In Taiwan, chewing BQ is a popular habit, particularly among males, with approximately 2 million regular users [13]. In a previous study, we found that male Taiwanese oral and pharyngeal cancer patients with a history of high-frequency BQ use had poor survival [14, 15].

By 2004, the International Agency for Research on Cancer (IARC) declared that BQ without tobacco is carcinogenic to humans (group 1) and increases the risk of oral cancer [16]. Although the masticatory practices and ingredients in BQ differ in different regions of the world, the AN is a major component of BQ worldwide, and the IARC has reported that AN alone is a group 1 carcinogen in humans. In the presence of slaked lime, the most abundant alkaloid of AN, arecoline, is hydrolyzed to arecaidine during the chewing process [17]. Arecoline has been shown to be cytotoxic to mammalian cells in vivo and in vitro [18–20]. In vitro studies the mutagenicity and genotoxicity of arecoline and arecaidine have been examined primarily in short-term experiments [21]. However, in carcinogenicity studies in animals, the IARC (2004) reported that evidence indicating that arecoline may cause cancer is limited, but inadequate evidence for the carcinogenicity of arecaidine [16].

In general, exposure to AN-derived carcinogens, particularly the alkaloids and the AN-derived *N*-nitrosamines, increases the risk of OPMDs and cancers of the oral cavity and pharynx in BQ chewers. Cytochrome P450 (*CYP*) enzymes are monooxygenases that catalyze many reactions involving carcinogens [22, 23]. During phase I metabolism, *CYP* families play important roles in detoxifying AN-derived compounds, such as arecoline [24], and are involved in the metabolic activation of arecoline-related *N*-nitrosamines [25]. A previous study suggested that arecaidine and three *N*-oxide metabolites are generated by the CYP enzyme system [26].

Environmental carcinogens and genetic polymorphisms, either separately or jointly, play an important role in the occurrence of oral and pharyngeal cancers. Environmental factors, such as alcohol use, BQ chewing, and cigarette smoking, were significantly associated with the risk of oral and pharyngeal cancers and OPMDs, and a synergistic effect among the use of these substances was also observed [6, 7, 16]. The interactions of environmental and genetic factors in the tumorigenesis of oral and pharyngeal cancers have been shown to be affected by various CYP enzyme-mediated metabolic processes [27–30].

Several studies have indicated that *CYP* polymorphisms affect the metabolism of tobacco-derived carcinogens and the risk of oral cancer [31–33]. However, reports of the risk of oral and pharyngeal cancers and OPMDs associated with AN-derived carcinogens are scant. Our review focuses on the role of the CYP enzyme-mediated metabolism in OPMDs and oral and pharyngeal cancers among BQ users and evaluates emerging data that potentially implicate arecoline- and arecoline-derived *N*-nitrosamines in tumorigenesis. The effects of *CYP* polymorphisms are worthy of investigation to further understand the role of genetic factors in susceptibility to OPMDS and cancers of the oral cavity and pharynx and to aid the development of prevention strategies for cancers related to BQ use.

## 2. AN-Derived *N*-Nitrosamines

### 2.1. Carcinogenicity of *N*-Nitrosamines In Vitro

The chewing of AN is believed to produce carcinogenic *N*-nitrosamines. Arecoline is the major compound of AN. The *N*-nitrosation of arecoline has been shown to form *N*-nitrosoguvacoline (NGL), 3-methylnitrosaminopropionaldehyde (MNPA), and 3-methylnitrosaminopropionitrile (MNPN) in vitro [34]. Based on studies of *Salmonella typhimurium* YG7108, CYP2A6 was found to be the most efficient activator of MNPN, followed by CYP1A1, and NGL was activated by CYP2A6. The genotoxicity of NGL was observed to be substantially lower than that of MNPN or MNPA [35]. Thus, that the human *CYP2A6* gene may play an important role in the mutagenic activation of AN-related *N*-nitrosamines has been suggested [35]. Studies on rodents have shown that MNPN, MNPA, and NGL are carcinogenic. In carcinogenicity studies on animals, the IARC (2004) determined that evidence of MNPN carcinogenicity is sufficient [16]. The carcinogenicity of MNPN may be caused by DNA methylation, which has been observed in rats treated with MNPN [21, 36].

### 2.2. Endogenous Nitrosation and *N*-Nitrosamines Carcinogenicity

Endogenous nitrosation occurs during BQ chewing, exposing BQ chewers to four *N*-nitrosamines derived from arecoline [37]. These AN (arecoline)-derived *N*-nitrosamines include MNPN, MNPA, NG, and *N*-nitrosoguvacine (NGC). These arecoline-derived *N*-nitrosamines are undetectable in the AN before chewing and are formed by the endogenous nitrosation of arecoline.  Table 1 lists the maximum levels of NGL (142 ng/mL), NGC (26.6 ng/mL), and MNPN (11.4 ng/mL) in the saliva during BQ chewing without tobacco.

Many BQ chewers often swallow the quid juice, which contains the precursors of the nitrosamines. The pH of stomach acid likely facilitates the nitrosation of secondary and tertiary amines from the quid. A modified *N*-nitrosoproline test showed that the urinary levels of *N*-nitrosoproline, an endogenous nitrosation marker, are 2.4- to 6.5-fold higher in BQ chewers, with or without tobacco, compared to nonchewers [42, 43]. Urinalysis of Syrian hamsters fed AN and a nitrite source detected NGL and its metabolite, *N*-nitrosonipecotic acid [44, 45], indicating that exposure to nitrosamine carcinogens formed by endogenous nitrosation is likely higher among BQ chewers who swallow the BQ juice [46]. Several case-control studies have also indicated that swallowing the BQ juice is associated with a significant increase in the risk of oral cancer [6, 47, 48].

### 2.3. *CYP1A1*-Mediated Metabolism of *N*-Nitrosamines

In a study of *S. typhimurium* YG7108, CYP1A1 was the second most efficient activator of MNPN, after CYP2A6, and MNPA activation was catalyzed to a lesser extent by CYP1A1 [35]. Previous studies have demonstrated that *CYP1A1* polymorphisms are associated with susceptibility to tobacco-related oral cancers [31–33, 49, 50]. Studies of the association between *CYP1A1* polymorphisms and BQ-related oral cancers are scant.

Kao et al. found that people with the *CYP1A1* Exon 7 polymorphism G/G genotype (val/val) are susceptible to BQ-related oral cancer and OPMDs [29]. They found that people who have the G/G and A/G (ile/val) genotype have significantly higher rates (*P* < .0001) of oral cancer (7.6% and 79.2%, resp.) and OPMDs (10% and 68.3%, resp.) than controls (1.4% and 53.4%, resp.). Kao et al. calculated odds ratios for the development of oral cancer of 18.86 and 5.08 for those with the G/G (95% CI, 3.61–98.52) or A/G (95% CI, 2.64–9.76) genotype of *CYP1A1*, respectively, and also reported odds ratios for the development of OPMDs of 15.23 and 2.67 for those with the G/G (95% CI, 2.76–83.98) or A/G (95% CI, 1.32–5.40) genotype, respectively. These novel findings indicated that people with the G (val) allele may have an earlier onset age of oral cancer [29]. Another study showed that people with the *CYP1A1* m2 polymorphism within the *Nco*I restriction site (−/−) or the *CYP1A1* m1 polymorphism at the *Msp*I site (+/−) and (−/−) had a significantly higher risk of oral submucous fibrosis (OR = 8.25; 95% CI, 4.31–15.80; OR = 2.88; 95% CI, 1.57–5.24; and OR = 3.16; 95% CI, 1.10–9.04, resp.) [51].

### 2.4. The *N*-Nitrosamine-Metabolizing *CYP2A6* Gene

Based on previous studies, we conclude that human *CYP2A* and *CYP2E* subfamily members play important roles in the metabolic activation of arecoline-related *N*-nitrosamines [52–54]. Located on human chromosome 19, the *CYP2A6* gene consists of 350 kilobases located at 19q 12–19q 13.2 [55–57]. Thirteen alleles of the *CYP2A6* gene have been identified (CYP2A6*1 through CYP2A6*11 and CYP2A6*1 × 2; Table 2). The CYP2A6*1 allele has 2 forms, CYP2A6*1A and CYP2A6*1B, that produce a gene conversion with the *CYP2A7* gene in the 3′-untranslated region [58] and exhibit similar enzyme activity [58]. The CYP2A6*2, CYP2A6*3, CYP2A6*5, CYP2A6*6, CYP2A6*7, CYP2A6*8, CYP2A6*9, CYP2A6*10, and CYP2A6*11 genetic variants contain a different point mutation. In addition, the CYP2A6*10 variant contributes to variations in CYP2A6*7 and CYP2A6*8. The existence of CYP2A6*3 has been debated, but a previous study indicated that the CYP2A6*3 genetic variant was the result of multiple *CYP2A6* and *CYP2A7* gene conversions [59]. The CYP2A6*4 is from a deletion in the *CYP2A6* gene. The CYP2A6*1 × 2 comprises a variation at 2 sites in the *CYP2A6* gene, and the CYP2A6*1B allele is caused by gene conversion in the 3′-untranslated region of *CYP2A7*.

The various alleles of *CYP2A* express at least 13 different isoenzymes, among which *CYP2A6* metabolically activates the *N*-alkylnitrosamines, *N*-nitrosonornicotine, and 4-(methylnitrosamino)-1-(3-pyridyl)-1-butanone, which have relatively long alkyl chains [62, 63]. Miyazaki et al. first reported that *CYP2A* subfamilies play important roles in the mutagenic activation of AN-derived *N*-nitrosamines [35]. The CYP2A6 P450 enzymes are the primary activators of MNPN. In Asia, the most common variant of *CYP2A6* is CYP2A6*4 (the *CYP2A6* deletion). The frequency of CYP2A6*4 is approximately 6.6% to 15.1% in the Chinese population. It is the most common genetic variant in the Japanese population, occurring at a frequency of 20.0% to 31.0% (Table 2).

People are classified as poor (PM), extensive (EM), or ultrarapid metabolizers (UM) based on their type of genetic variation [64]. The UMs have 2 active alleles of the *CYP2A6* gene, including the CYP2A6*1 × 2 variant. Phenotyping assays have indicated that 2 or more copies of active *CYP2A6* alleles may result in a rapider nicotine metabolism. People with 1 or 2 copies of active gene alleles, such as CYP2A6*1/*1, are extensive metabolizers [60, 65], whereas PMs are those with null alleles, such as CYP2A6*2/*2 or CYP2A6*4/*4, with no enzyme function or less activity regarding probe substrates. People who are homozygous for the CYP2A6*2 allele have little coumarin-hydroxylation activity (<0.1%) [60, 61], and EMs exhibit low activity (<15%) when nicotine is used as the probe substrate [58, 60, 66, 67].

Previous reports have indicated that the deletion of *CYP2A6* (CYP2A6*4C) may reduce the risk of lung cancer [68–71], suggesting that people with CYP2A6*4C may not activate tobacco nitrosamines from smoking. Because a genetic variation within the *CYP2A6* gene appears to reduce xenobiotic activation, *CYP2A6* polymorphisms may also reduce the metabolic activation of AN-derived nitrosamines. In Sri Lanka, a study found that the deletion polymorphism, CYP2A6*4C/*4C, reduces susceptibility to oral squamous cell carcinoma (OR = 0.14; 95% CI, 0.03–0.72) among habitual BQ chewers with oral lesions, suggesting that BQ chewers with reduced *CYP2A6* activity because of polymorphisms may be at lower risk for oral cancer [30].

### 2.5. The *N*-Nitrosamine-Metabolizing *CYP2E* Gene

The *CYP2E* subfamilies include *CYP2E1* and *CYP2E2*. The *CYP2E1* gene has been shown to be the primary activator of *N*-nitrosodimethylamine and *N*-nitrosodiethylamine, which are *N*-nitrosamines with relatively short alkyl chains [62, 63]. A case-control study of 41 male oral cancer patients and 123 healthy controls found that people with the *CYP2E1* c1/c2 or c2/c2 genotype had a higher risk of oral cancer (multicovariate-adjusted OR = 2.0; 95% CI, 0.8–5.4) than those with the c1/c1 genotype [27]. However, the association was not statistically significant. Hung et al. also reported a significant association between *CYP2E1* polymorphisms and oral cancer among those without BQ-chewing habits (OR = 4.7; 95% CI, 1.1–20.2), but not among BQ chewers [27]. All BQ chewers were also cigarette smokers, and the chewing habits had a significant effect on the risk of oral cancer. Thus, Hung et al. suggested that the risk of oral cancer associated with combined habits of BQ chewing and smoking may be too striking to have modified effects by the *CYP2E1* genotype. A more recent study showed that polymorphisms in *CYP2E1* within the PstI site (+/−) were significantly associated with oral submucous fibrosis (OR = 3.14; 95% CI, 1.14–8.62) [51].

### 2.6. Arecoline-Induced, *CYP26B1*-Mediated Retinoic Acid Metabolism

In a previous study, we treated normal human gingival fibroblasts (HGFs) with arecoline and screened for the presence of a novel *CYP26B1* by using a microarray [72]. The expression of CYP26B1 was subsequently confirmed using quantitative reverse transcription and real-time polymerase chain reaction [48]. The *CYP26B1* gene is located at the human 2p13.2 region and may play an important role in variations in retinoic acid (RA) metabolism associated with oral lesions. Hyperkeratosis and hyperplasia of the oral mucosa can be caused by insufficient retinol intake [73], and the findings of previous studies have indicated that remission of oral leukoplakia in BQ chewers treated with RA may result from the suppression of AN-related metabolism, rather than the inhibition of tumorigenesis [74–76]. Because it is a critical regulator of cell proliferation, cell differentiation, and apoptosis, RA deficiency may play an important role in carcinogenesis [73, 77–79].

At an RA concentration of 100 *µ*g/mL, the repression level of *CYP26B1* was approximately 15-fold in cultured primary HGFs obtained from a healthy volunteer in our previous study [72]. We suggested that *CYP26B1* may be involved in detoxification processes, and arecoline treatment in normal HGFs was shown to repress susceptibility [72]. We detected higher levels of CYP26B1 mRNA and protein expression in human oral cancer cells compared with adjacent noncancerous tissues. The findings of another previous study suggested that CYP26B1 mRNA is predominantly expressed in the adult human cerebellum and may be associated with the protection of specific human tissues from exposure to RA [80]. We found that the *CYP26B1* polymorphism AA significantly correlated with the risk of oral cancer (OR = 2.26; 95% CI, 1.35–3.80), and BQ chewers with the AA genotype had a significantly increased risk of oral cancer (OR = 70.04; 95% CI, 13.62–360.11). We concluded that *CYP26B1* is a novel candidate gene in the development of BQ-related oral cancer and speculated that *CYP26B1* may be involved in the metabolism of arecoline-related compounds [48]. 

In the oral mucosa of BQ chewers, *CYP26B1* induction alters the RA metabolism [79]. A previous study demonstrated that *CYP26* members may play a role in RA accumulation in human epidermal keratinocytes [81]. Klaassen et al. [82] found higher levels of RA-inducible CYP26 mRNA expression and higher RA turnover rates in oral squamous cell carcinoma cell lines, compared with noncancerous oral KB cells, and that oral KB cells from cancer patients exhibit a 15-fold higher RA turnover rate compared with noncancerous oral KB cells [83].

A previous study indicated that high-level CYP26 expression may be associated with head and neck cancer [82]. The expression of CYP26 was induced through an RA-receptor-mediated mechanism in breast and colon carcinoma cells [84]. A higher expression of CYP26 caused intracellular RA depletion in Barrett-associated adenocarcinoma [78], whereas other studies have indicated that the expression of CYP26 is downregulated in noncancerous human epidermis [85, 86]. These findings indicate that the RA metabolism is an important factor in the development of oral cancer.

## 3. Discussion

Oral cancer is one of the most common cancers worldwide and was ranked the eighth leading cause of cancer-related deaths in 2000 [1]. Incidence rates of 8.8 and 5.1 per 100 000 cases and mortality rates of 7.3 and 3.2 per 100 000 cases have been reported for oral cancer among males and females, respectively [1]. In Taiwan, BQ production has increased approximately 44-fold [16]. An international study found that Taiwan had the fourth highest prevalence of oral and pharyngeal cancers, preceded by Papua New Guinea, the Solomon Islands, and Sri Lanka [2]. In BQ-endemic areas, oral and pharyngeal cancers and the most common OPMDs (such as OSF and oral leukoplakia) appear to be associated with BQ use, whereas cigarette smoking and heavy alcohol drinking are the major risk factors in western countries.

**Figure 1:**
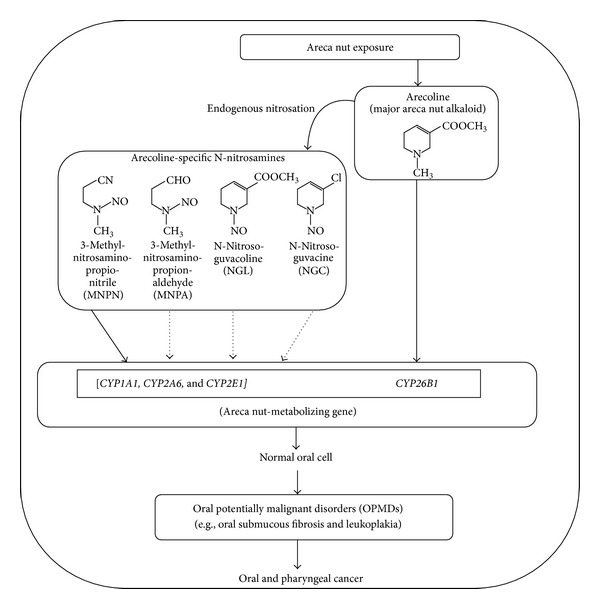
Simplified flow chart for postulated main effects of areca nut induced oral carcinogenesis *via* cytochrome P450 (*CYP*) gene.

Numerous genes are involved in carcinogen metabolism, and most studies have found that *CYP* polymorphisms affect the risk of oral cancer associated with variations in the metabolism of tobacco-derived carcinogens. Arecoline, arecaidine, and other BQ-related metabolites may exist at nanogram concentrations in human blood, and the level of arecoline is significantly associated with the quantity of BQ used [87]. We summarized the findings of studies of the effects of *CYP* polymorphisms on BQ chewing-related symptoms, including cancer of the oral cavity and pharynx and OPMDs, in Table 3. Among xenobiotic metabolizing enzymes, *CYP1A1*, *CYP2A6*, and *CYP2E1* may activate AN-derived nitrosamines. In addition, the expression of CYP26B1 may be induced by arecoline and may be related to RA metabolism. The flowchart in Figure 1 depicts the main effects of *CYP* genetic variants on AN-induced carcinogenesis.

To the best of our knowledge, only 2 studies have reported a relationship between the genetic polymorphism of *CYP1A1* and the risk of oral cancer and OPMDs [29, 51]. Higher-risk patients with the *CYP1A1* G (val) allele should be strongly encouraged to avoid BQ use and maintain good oral hygiene. People with the CYP2A6*4C/*4C genetic variant may be at lower risk of oral cancer because their genotype suppresses the activation of AN-related procarcinogens [30]. The *CYP2E1* polymorphisms may increase the risk of oral cancer [27, 51]. In addition, the findings of our previous study suggest that the combination of higher CYP26B1 expression and polymorphism is associated with an increased risk of oral cancer [48].

Future studies on *CYP26B1* antagonists may identify novel RA-related methods of chemoprevention or treatment for OPMDs. Patients with high-risk alleles who chew BQ have an elevated risk for oral and pharyngeal cancers, and the risk is particularly high for OPMDs patients. We propose that the high-risk genotypes, such as *CYP1A1*, *CYP2A6*, *CYP2E1*, and *CYP26B1*, should be targeted for the development of a single-nucleotide polymorphism (SNP) gene chip for risk assessment in OPMDs patients, especially for those with BQ chewing habits.

## 4. Conclusion

The accumulation of such findings will be useful for the identification of high-risk patients and the development of novel therapeutic strategies for blocking the activation of AN-related compounds and targeting the *CYP* gene. Large-scale studies on the polymorphisms of *CYP* genes in BQ chewers and the genetic variants related to oral and pharyngeal cancers or OPMDs are warranted. The identification of molecular mechanisms elucidated by future pharmacogenomics studies will establish a strong foundation for the development of chemoprevention strategies, SNP-based clinical diagnostic tools (e.g., specific SNP barcodes for BQ-associated oral disorders), and effective treatments for BQ-related oral disorders.

## Figures and Tables

**Table 1:** Detected saliva levels (ng/mL) of nitrosamines such as MNPN, NGL, and NGC in chewers with tobacco and without tobacco.

BQ-specific *N*-nitrosamines	BQ alone (without tobacco)	BQ + tobacco	References
MNPN	0.5–11.4	—^a^	Prokopczyk et al., 1987 [36]
NGL	0–5.9	0–7.1	Nair et al., 1985 [38]
	0.6–8.8	3.1–23.5	Nair et al., 1987 [39]
	2.2–9.5^b^	4.3–45^b^	Wenke et al., 1984 [40]
	0–142	—	Stich, 1986 [41]
NGC	0–26.6	0–30.4	Nair et al., 1985 [38]

Adapted from [16].

BQ: betel quid.

^a^The data not reported.

^b^In ppb.

**Table 2:** The nomenclature of *CYP2A6* and allele frequencies in population.

Allele	Frequencies in population							Nucleotide change	Effect	Enzyme activity	
	Caucasian (%)	African American (%)	Swedes (%)	Finns (%)	Spaniards (%)	Chinese (%)	Japanese (%)			In vitro	In vivo
CYP2A6∗1A	66.5	—^a^	98.9	98.6	97.0	43.2	40.0–42.0	None	—	Normal	Normal
CYP2A6∗1B	30.0	—	—	—	—	40.6	38.0–41.0	Gene conversion at 3′-flanking region	—	—	—
CYP2A6∗1 × 2	0.7	—	—	—	—	0.4	0.0	—	Duplication of CYP2A6	—	—
CYP2A6∗2	1.1–3.0	0.3	1.1	1.4	3.0	0.0–0.7	0.0	488 T→A	L160H	None	None
CYP2A6∗3	—	—	—	—	—	—	—	CYP2A6/CYP2A7 hybrid	—	—	—
CYP2A6∗4A	0.5–4.9	—	—	—	—	6.6–15.1	20.0–31.0	CYP2A6 deletion	CYP2A6 deletion	—	None
CYP2A6∗4B	—	—	—	—	—	—	—	CYP2A6 deletion	CYP2A6 deletion	—	None
CYP2A6∗4C	—	—	—	—	—	—	—	—	—	—	—
CYP2A6∗4D	—	—	—	—	—	—	—	CYP2A6 deletion	CYP2A6 deletion	—	None
CYP2A6∗5	0.0–0.2	—	—	—	—	1.0	0.0	1436 G→T	G479V	None	None
CYP2A6∗6	—	—	—	—	—	—	0.4	383 G→A	R128Q	Down	—
CYP2A6∗7	1.0	—	—	—	—	2.2	6.3	1412 T→C; gene conversion at the 3′-flanking region	I471T	Down	Down
CYP2A6∗8	0.0	—	—	—	—	3.5	1.6	1454 G→T; gene conversion at the 3′-flanking region	R485L	—	Normal
CYP2A6∗9	5.2	—	—	—	—	15.7	—	—48 T→G	TATA box	Down	—
CYP2A6∗10	0.0	—	—	—	—	0.4	1.6	1412 T→C; 1454 G→T; gene conversion at the 3′-flanking region	I471T; R485L	—	Down
CYP2A6∗11	—	—	—	—	—	—	—	670 T?C	S224P	Down	Down

Adapted from [57, 60, 61].

^a^The data not reported.

**Table 3:** The association studies between cytochrome P450 (*CYP*) polymorphism and betel quid-related oral disorders.

*CYP * gene	Cases/number Controls/ number	Chewing habit of cases/controls	OR (95% CI)	Conclusion	Population/ reference
*CYP1A1 *	Oral cancer/106 Controls/146	BQ, 62.3%/15.0%	Gene effects: Exon 7 A/G (ile/val) A/G versus A/A, 5.08 (2.64–9.76)* G/G versus A*/*A, 18.86 (3.61–98.52)*	Subjects with* CYP1A1* carrying G allele increased the risk for OPMDs and oral cancer	Taiwan/[29]
	OPMDs/60 Controls/146	BQ, 75.0%/15.0%	Gene effects: Exon 7 A/G (ile/val) A/G versus A/A, 2.67 (1.32–5.40)* G/G versus A*/*A, 15.23 (2.76–83.98)*		
			Gene effects: 3′UTR *Msp*I site	No significant association	
	OSF/75 Controls/150		Gene effects: m1 at *Msp*I site (+/−) versus (+/+), 2.88 (1.57–5.24)* (−/−) versus (+/+), 3.16 (1.10–9.04)* Gene effects: m2 at *Nco*I site (−/−) versus (+/+), 8.25 (4.31–15.80)*	Subjects with *CYP1A1* polymorphisms had significantly increased risks of OSF	India/[51]
*CYP2A6 *	Oral lesions/286 (15 oral cancer, 62 OSF and 209 leukoplakia) Controls/135	betel, 100%/100%	Gene effects *1B/*4C versus *1A/*1A, 0.21 (0.05–0.88)* *4C/*4C versus *1A/*1A, 0.14 (0.03–0.72)*	BQ chewers with activity deficient of *CYP2A6* deletion decreased the risk of oral cancer	Sri Lanka/[30]
*CYP2E1 *	Oral cancer/41 Controls/123	BQ, 73.2%/12.2%	Gene effects c1/c2 + c2/c2 versus c1/c1, 2.0 (0.8–5.4) Among nonchewers c1/c2 + c2/c2 versus c1/c1, 4.7 (1.1–20.2)* Among chewers c1/c2 + c2/c2 versus c1/c1, 0.8 (0.2–3.3)	A significant relationship between *CYP2E1* polymorphisms and oral cancer risk was found among non-BQ chewers	Taiwan/[27]
	Oral cancer/106 Controls/146		Gene effects: at *Pst*I site (+/−) versus (+/+), 3.14 (1.14–8.62)*	Individuals with *CYP2E1 *at* Pst*I site polymorphism (+/−) may confer a significantly increased risk for oral cancer	India/[51]
*CYP26B1 *	Oral cancer/247 Controls/338	BQ, 85.4%/22.5%	rs707718 Gene effects A versus C, 1.48 (1.16–1.87)* AA versus CC, 2.26 (1.35–3.80)* Gene-BQ (+/−) interplay AA-BQ (+) versus CC-BQ (−), 70.04 (13.62–360.11)*	BQ chewing interacted with *CYP26B1*-AA significantly increased the risk of oral cancer	Taiwan/[48]
			Gene effects rs2241057, rs2286965, rs3768641	No significant findings	

OPMDs: oral potentially malignant disorders; OSF: oral submucous fibrosis; betel: betel quid chewing with or without tobacco; BQ: betel quid without tobacco; OR: odds ratios; CI: confidence interval; *statistical significance.

## Abbreviations

OPMDs: Oral potentially malignant disorders
BQ: Betel quid
AN: Areca nut
NGL: *N*-Nitrosoguvacoline
MNPA: 3-Methylnitrosaminopropionaldehyde
MNPN: 3-(Methylnitrosamino)propionitrile
NGC: *N*-Nitrosoguvacine
RA: Retinoid acid.
